# Exome sequencing in an Italian family with Alzheimer’s disease points to a role for seizure-related gene 6 (*SEZ6*) rare variant R615H

**DOI:** 10.1186/s13195-018-0435-2

**Published:** 2018-10-12

**Authors:** Lara Paracchini, Luca Beltrame, Lucia Boeri, Federica Fusco, Paolo Caffarra, Sergio Marchini, Diego Albani, Gianluigi Forloni

**Affiliations:** 1 Department of Oncology, Istituto di Ricerche Farmacologiche Mario Negri IRCCS, Via La Masa 19, 20156 Milan, Italy; 2 Dipartimento di Chimica, Materiali e Ingegneria Chimica “G. Natta”, Politecnico di Milano, Piazza Leonardo da Vinci 32, 20133 Milan, Italy; 3 Department of Neuroscience, Istituto di Ricerche Farmacologiche Mario Negri IRCCS, Via La Masa 19, 20156 Milan, Italy; 4 Department of Neuroscience, Istituto di Neurologia, Università di Parma, Via Gramsci 14, 43100 Parma, Italy

**Keywords:** Alzheimer’s disease, *SEZ6*, Exome sequencing, Rare variants

## Abstract

**Background:**

The typical familial form of Alzheimer’s disease (FAD) accounts for about 5% of total Alzheimer’s disease (AD) cases. Presenilins (*PSEN1* and *PSEN2*) and amyloid-β (A4) precursor protein (*APP*) genes carry all reported FAD-linked mutations. However, other genetic loci may be involved in AD. For instance, seizure-related gene 6 (*SEZ6)* has been reported in brain development and psychiatric disorders and is differentially expressed in the cerebrospinal fluid of AD cases.

**Methods:**

We describe a targeted exome sequencing analysis of a large Italian kindred with AD, negative for *PSEN* and *APP* variants, that indicated the *SEZ6* heterozygous mutation R615H is associated with the pathology.

**Results:**

We overexpressed R615H mutation in H4-SW cells, finding a reduction of amyloid peptide Aβ(1–42). *Sez6* expression decreased with age in a mouse model of AD (3xTG-AD), but independently from transgene expression.

**Conclusions:**

These results support a role of exome sequencing for disease-associated variant discovery and reinforce available data on *SEZ6* in AD models.

**Electronic supplementary material:**

The online version of this article (10.1186/s13195-018-0435-2) contains supplementary material, which is available to authorized users.

## Background

Alzheimer’s disease (AD) is a multifactorial neurodegenerative disorder whose onset is mostly sporadic [1]. The genetic background has a major role in AD, and DNA variants may contribute, ranging from predisposing risk factors (having from medium to large effect size, such as the ε4 allele of the *APOE* gene) [2] to full penetrant causal mutations in a few genes, namely presenilins (*PSEN1* and *PSEN2*) and the amyloid-β (A4) precursor protein (*APP*) [3, 4]. *PSEN1/2* and *APP* gene mutations have been linked to early-onset, autosomal dominant familial forms of Alzheimer’s disease (FAD) [5, 6]. Recently, large-scale whole-exome sequencing has found rare variants reported to contribute to AD risk, such as in the *PLCG2*, *ABI3*, and *TREM2* genes [7]. These findings indicate the involvement in familiar forms of AD of variants belonging to genes other than *PSEN1/2* and *APP*, which may have a causal or predisposing role, as recently reported for *SORL1* gene [8].

We report an Italian family with several cases of AD (having an onset between 60 and 70 years) negative for *PSEN1/2* or *APP* mutations and whose available affected members were found to bear *SEZ6* gene rare missense variant R615H. We describe the genetic, *in vitro*, and *in vivo* findings further supporting a role for *SEZ6* in AD molecular mechanisms.

## Methods

### Family and patient description

**Fig. 1:**
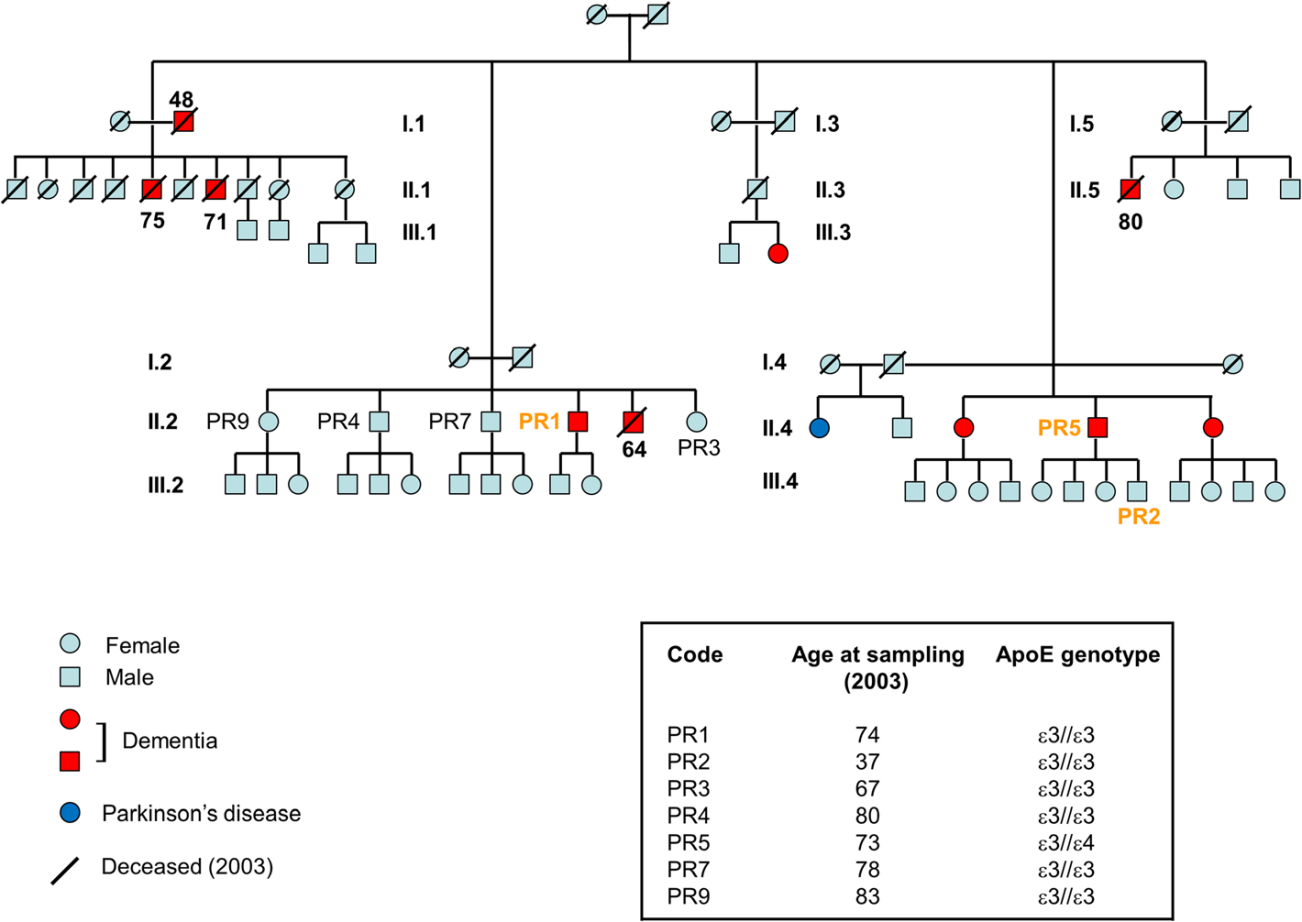
Pedigree of the Italian family with Alzheimer’s disease. We report clinical information for the last three generations after the founders. Sex, age at sampling, and apolipoprotein E (*APOE*) genotype of each available family member indicated in the box. The numbers next to subjects with dementia are the age at death. The roman numbers refer to the generation, with the progressive numbers linking to every generation sibling

The family’s pedigree is reported in Fig. 1. We extracted DNA for exome sequencing analysis from the members indicated by the code PR (seven subjects). We had clinical details about three generations after the founder. Ten dementia cases were reported in the whole pedigree, with an additional member having Parkinson’s disease. The age of onset of neurodegenerative disorders ranged from 60 to 70 years. In the first generation, one early-onset dementia case was reported (age at death, 48 years). In the second generation, 8 of 25 siblings (32%) were diagnosed with AD, with an additional case in the third generation (age at onset 64 years). The remaining siblings of this generation were cognitively normal, aged between 35 and 45 years. Apolipoprotein E genotype (*APOE*) of available patients was in all cases ε3//ε3 apart from PR5 (ε3//ε4). Two siblings of PR5, diagnosed with AD, had dementia too, but they were unavailable for sampling.

Sporadic AD cases (*n* = 9) and cognitively normal elderly control subjects (*n* = 191) were included for independent evaluation of the *SEZ6*(R615H) variant frequency by digital droplet PCR (ddPCR).

Patients and healthy control subjects were recruited by the same clinical center, and AD was diagnosed according to international criteria. Healthy control subjects were spouses of patients coming to clinical attention, and they had no sign of neurodegenerative disorders and Mini Mental State Examination (MMSE) scores in the normal range [9].

### Exome sequencing and *APOE* genotyping

The full-exome sequencing of 4811 disease-associated genes (clinical exome) was done starting from 50 ng of DNA diluted in Tris-HCl 10 mM, pH 8.5 (TruSight One Sequencing Panel; Illumina, San Diego, CA, USA), following the manufacturer’s instructions. Briefly, capture-based libraries were prepared by pooling three samples per time. The libraries’ concentrations were calculated using a Qubit® dsDNA High-Sensitivity Assay Kit (Invitrogen, Carlsbad, CA, USA), and the distribution of DNA fragments for each library was evaluated using a high-sensitivity DNA kit and a 2100 Bioanalyzer (Agilent Technologies, Santa Clara, CA, USA). Each library was run on a MiSeq platform (Illumina) using a 2 × 150-bp (300 cycles) configuration on a V3 sequencing flow cell.

Data analysis was performed according to best practice from the bioinformatics community. Raw sequence fragments (reads) were aligned to the reference genome (human, build hg19) with the Burrows-Wheeler alignment tool [10], followed by post-processing to recalibrate base call quality scores. Variants were called with the Genome Analysis Toolkit [11–13], using the HaplotypeCaller method, then annotated with the Variant Effect Predictor [14] and loaded into a specialized database [15] for further analysis. *In silico* mutation impact predictions were extracted from the dbNSFP database [16]. For computation, we used the “bcbio” pipeline (https://github.com/chapmanb/bcbio-nextgen) running on a high-performance computing platform as part of the Cloud4CaRE project. Data files were uploaded to the European Nucleotide Archive with accession number pending.

Selection of candidate variants used the following criteria: (a) depth at least 30×; (b) low frequency in the general population (< 1% in the 1000 Genomes Project); (c) at least a damaging predicted effect as reported from the dbNSFP; and (d) present in all family members affected by AD or their offspring. The *APOE* genotype was assessed by restriction fragment length polymorphism using the CfoI (Roche, Basel, Switzerland) restriction enzyme, as previously described [17].

### Exome sequencing validation by digital droplet PCR

ddPCR experiments were done with the Bio-Rad QX200TM ddPCR system (Bio-Rad Laboratories, Hercules, CA, USA). The mutational assay for *SEZ6* R615H was carried out according to the manufacturer’s instructions. Briefly, the TaqMan™ reaction mix, composed of 2× ddPCR Supermix for probes (no deoxyuridine triphosphate), 20× custom target probes for mut SEZ6 (probe sequence: CTACGG**TCA**TGGGCAG-FAM), and 20× reference probes for wild-type SEZ6 (probe sequence: CTACGG**TCG**TGGGCA-HEX), was assembled at a final concentration of 450 nM and 20 ng of DNA in a volume of 20 μl. This reaction mix was added to a DG8 cartridge together with 60 μl of droplet generation oil for probe and used for droplet generation (QX200 droplet generator; Bio-Rad Laboratories). Droplets were then manually transferred to 96-well PCR plates and placed on a thermal cycler (T100 Thermal Cycler; Bio-Rad Laboratories) for the PCR amplification (thermal cycling conditions: 95 °C for 5 min, 95 °C for 30 s, and 55 °C for 1 min, 40 cycles; 98 °C for 10 min and 4 °C infinite; ramping rate 2 °C/s). The PCR plate was then transferred into the QX100 Droplet Reader for the fluorescence measurement of FAM and HEX probes. The numbers of positive and negative droplets were used to calculate the concentrations (copies/μl) of the target and the reference *SEZ6* DNA sequence and their Poisson-based 95% CIs, excluding reactions with fewer than 10,000 total events (positive and negative) (QuantaSoft Analysis pro software 1.0.596; Bio-Rad Laboratories).

For family members and patients with sporadic AD, experiments were run in duplicate; the assay on the healthy population was run once.

### Cloning and overexpression of *SEZ6*(R615H) in H4-SW cells

#### pSEZ6(R615H) cloning

Synthetic *SEZ6*(R615H) complementary DNA was provided by GenScript® in pCDNA3.1(+) vector and expanded in competent *Escherichia coli* cells (strain JM109; Promega, Madison, WI, USA). After purification, p*SEZ6*(R615H) was verified through the unique enzymatic restriction site PmeI (New England Biolabs, Hitchin, UK) and agarose gel electrophoresis.

#### Cell culture

H4-SW neuroglioma cells overexpressing human *APP* gene harboring the Swedish (SW) mutation [18] were grown in DMEM supplemented with 10% FBS, 2 mM l-glutamine, and antibiotics (100 U/ml penicillin, 100 μg/ml streptomycin, 300 μg/ml hygromycin B, 10 μg/ml blasticidin-S).

Transient transfection was done using FuGENE® HD Transfection Reagent (Promega), and cells were selected with G418 (1200 μg/ml) after 48 h. For clonal selection of *SEZ6*(R615H) mutants, we picked colonies and analyzed DNA and protein extracts by PCR and Western blotting. Finally, a single-point mutation (G→A) leading to R615H substitution was checked by Sanger sequencing.

### PCR for *SEZ6*(R615H) expression in H4-SW cells

PCR was run in a 20-μl mixture containing 50 ng of DNA, 0.5 mM each of forward primer 5′-CTACGGTCATGGGCAGGATTG-3′, which contains the single-point mutation (G→A), and the reverse oligonucleotide primer 5′- ATCATGGCAGGTGAGGATGGACT-3′ (metabion, Planegg, Germany); 1× PCR buffer 200 mM Tris-HCl, 500 mM KCl (Thermo Fisher Scientific, Waltham, MA, USA); 2.5 mM deoxynucleotide triphosphate (Thermo Fisher Scientific); 25 mM MgCl_2_ (Thermo Fisher Scientific); and 1 unit of Taq polymerase (Thermo Fisher Scientific). Amplification was done with an initial denaturation at 95 °C for 2 min, followed by 30 cycles of denaturation at 95 °C for 30 s, annealing at 61.7 °C for 30 s, extension at 72 °C for 70 s, and a final 5-min extension at 72 °C. The resulting PCR fragments were resolved by 1% agarose gel electrophoresis (Sigma-Aldrich, St. Louis, MO, USA).

### Western blotting for SEZ6 overexpression in H4-SW cells

To assess protein overexpression of *SEZ6* in H4-SW, protein extracts (18 μg) were separated on 8% SDS-PAGE gel and transferred to a nitrocellulose membrane. Blots were developed using horseradish peroxidase-conjugated secondary antibodies and the ECL chemiluminescence system (MerckMillipore, Burlington, MA, USA). All blots were normalized to α-tubulin and quantified using ImageJ software (National Institutes of Health, Bethesda, MD, USA). The following antibodies were used: anti-α-tubulin (1:7500; Abcam, Cambridge, UK) and anti-SEZ6 (1:1000; Aviva Systems Biology, San Diego, CA, USA).

### DNA sequencing

To verify the presence of the single point mutation, we amplified the region containing the mutated base by PCR with forward primer 5′- GAGATCACAGACTCGGCTG-3′ and the reverse primer 5′- ATCATGGCAGGTGAGGATGGACT-3′ (metabion). The total amount of the generated PCR product was purified using the Wizard SV Gel PCR Clean-Up System (Promega) and sent to a Sanger sequencing service (Eurofins Genomics, Ebersberg, Germany). Output data were analyzed using Chromas Lite 2.01 software.

### Aβ(1–42) and Aβ(1–40) in H4-SW cells expressing *SEZ6*(R615H)

A specific sandwich enzyme-linked immunosorbent assay (ELISA) (Immuno-Biological Laboratories Co., Gunma, Japan) was used to measure Aβ(1–42) and Aβ(1–40) concentrations in conditioned media from cultured H4-SW cells. A total of 150 × 10^3^ cells were seeded in a six-well plate and grown overnight. The next day, the medium was changed, and after 48 h it was collected and immediately frozen after the addition of a broad-spectrum protease inhibitor (Sigma-Aldrich). An aliquot of 100 μl was used for ELISA to assess each value in triplicate.

### Western blot analysis for *Sez6* brain expression in 3xTG-AD mice

For *Sez6* brain expression analysis, we used 3xTG-AD mice at 3, 9, and 19 months of age. This triple-transgenic model harbors human *PS1*(M146 V), *APP*(SW), and MAPT(P301L) transgenes, and starting from around 9 months of age, mice develop at brain level amyloid plaques and protein tau tangles. They also show early signs of synaptic dysfunction (starting from around 3 months of age), including long-term potentiation alteration [19]. Strain, age, and sex-matched nontransgenic animals were used as controls. Mice were housed at 23 °C room temperature with food and water *ad libitum* and a 12-h/12-h light/dark cycle. To obtain brain protein extract, the cortex was dissected from a single brain hemisphere and homogenized with ice-cold lysis buffer (pH 7.4) containing 1% Triton X-100 and a broad-range protease inhibitor cocktail. Cortex protein extract (20 μg) was analyzed as described above.

### Statistics

Data analysis was done using Prism® version 6.0 software (GraphPad Software, La Jolla, CA, USA). In vitro and in vivo data were compared using one-way analysis of variance followed by Tukey’s post hoc test. Two-tailed levels of significance were used, and *p* < 0.05 was considered significant.

## Results

### Exome sequencing and *APOE* genotyping

To identify variants linked to dementia phenotype, we sequenced DNA samples from family members (healthy and AD cases) and unrelated patients with sporadic AD for a set of over 4000 genes reported as implicated in rare and genetic diseases. Our initial analysis identified 15,745 variants passing our quality control filters (variant depth 30× or more). Many of these were common polymorphisms present in the general population, so we selected only those rare in the European population (< 1% frequency), lowering the count to 612 (Additional file 1: Table S1).

To further narrow the search for variants of interest, we used *in silico* analysis to restrict our findings to those predicted as damaging for protein, finding 138 variants (Additional file 1: Table S1). The majority (96.4%) of possible damaging variants were common between both familial and sporadic AD samples. On the contrary, five variants (3.6%) were exclusive to the family samples (Table 1). In particular, a missense variant in the *SEZ6* neuronal gene (c.1844G>A, R615H) was present only in the two available AD cases (PR1 and PR5) and in a first-degree relative (PR2, son of PR5). This variant was localized on one of the extracellular CUB domains of the protein [20, 21] and was predicted to have a high damaging potential (Combined Annotation Dependent Domain [CADD] score = 23). This prompted us to further focus on this variant.

**Table 1:** Variants exclusive of family members and satisfying the filtering criteria

Chr	Position	Gene	Variant	Amino acid change	(%)	dbSNP ID	Found in (family code)
chr8	144,589,984	ZC3H3	c.1646 C > T	p.Ser549 Leu	0.5%	rs 149,025,999	*PR 1*, PR 2, PR 3, PR 4, *PR 5*, PR 7, PR 9
chr9	738,341	KANK1	c.3391 G > C	p.Ala1131Pro	0.1%	rs 180,816,986	*PR1*, PR3, *PR5*
chr17	27,286,417	**SEZ6**	c.1844 G > A	p.Arg615 His	0.01%	rs 371,753,097	*PR1*, PR2, *PR5*
chr20	57,598,807	TUBB1	c.326 G > A	p.Gly109 Glu	0.2%	rs 41,303,899	*PR1*, PR2, PR3, *PR5*
chr22	24,717,509	SPECC1L	c.562 C > T	p.Leu188 Phe	0.9%	rs 56,168,869	*PR1*, PR2, PR3, *PR5*, PR9

*Chr* Chromosome number

Percentage population frequency refers to data of the European population frequency derived from the 1000 Genomes Project at the time the manuscript was written. *See* the “Methods” section of text for further details. Chromosome positions refer to the hg19 assembly. The gene of interest (SEZ6) is highlighted in bold, and members affected with AD are Italic

### Validation of exome sequencing *SEZ6*(R615H) data and variant screening in sporadic AD cases and healthy control subjects

Because the clinical exome results indicated a mutation in *SEZ6* gene (c.1844G>A) as unique to the available family members with AD, we performed an independent validation to confirm the result. Using ddPCR, we tested for the *SEZ6* variant in exome sequencing-positive family members (*n* = 3) and in sporadic AD cases (*n* = 9). To exclude the possibility that the polymorphic variant of *SEZ6* identified could be detected at low frequency in the healthy population, too, the mutational assay was also done in a control group of 191 cognitively healthy people.

**Fig. 2:**
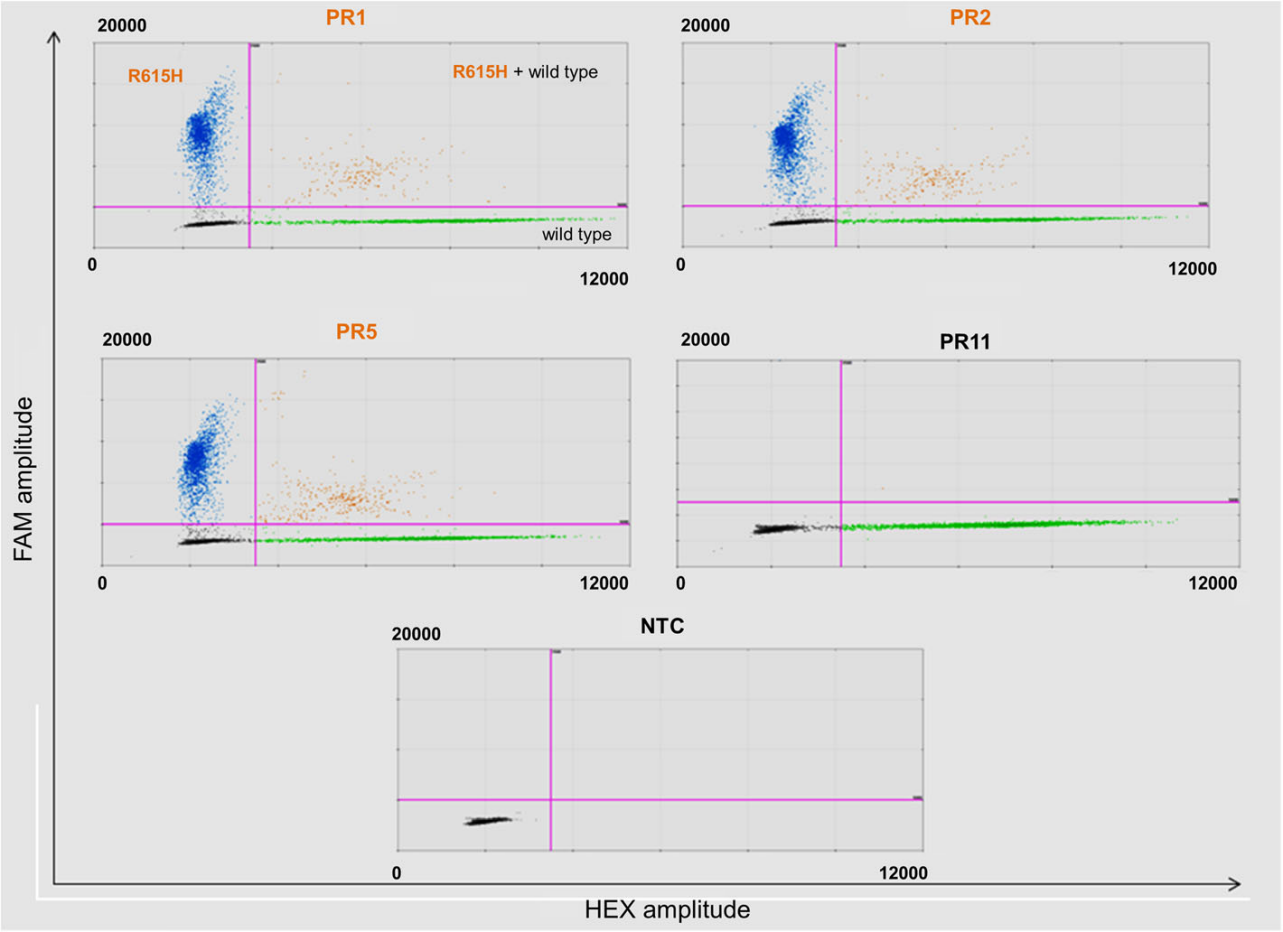
Digital droplet PCR validation of the exome sequencing data. For each patient, a 2D dot plot is shown, reporting the distribution of fluorescence (on the *y*-axis FAM amplitude, and on the *x*-axis HEX amplitude). FAM and HEX are the fluorescent dyes for the *SEZ6* mutant and *SEZ6* wild type, respectively. On the basis of the fluorescence measurements and the droplet distributions, thresholds (*pink lines*) were set to 5000 for the FAM channel (*y*-axis) and 3000 for the HEX channel (*x*-axis). Negative droplets (*gray*), FAM-positive (*blue*), HEX-positive (*green*), and FAM/HEX double-positive (*orange*) droplets are reported for the four cases and no-template control (NTC) analyzed. Each case represents the sum of independent reactions

Figure 2 shows *SEZ6* mutational analysis of three family members (PR1, PR2, and PR5) and a representative case of sporadic AD (PR11). Wild-type *SEZ6* (green droplets) was detected in all samples, whereas mutated *SEZ6* (blue) was detected only in the PR1, PR2 and PR5 samples. A single event with both wild-type and mutated *SEZ6* was detected in PR11, probably a polymerase artifact.

Regarding a quantitative measure of the *SEZ6* variant, Table 2 reports the concentration as the number of target molecules/μl of wild-type and mutant *SEZ6* in all sporadic cases (*n* = 9), in family members (*n* = 3), and in healthy individuals (*n* = 191). Wild-type *SEZ6* copies were detected in all groups. The means of wild-type *SEZ6* copies/μl were 564, 258, and 130 in the healthy control group, sporadic AD cases, and family members, respectively. A high concentration of mutant *SEZ6* was detected in family member samples. The simultaneous presence of the wild-type and the mutated form of *SEZ6*, with ratios (mutated *SEZ6* to wild-type *SEZ6*) ranging from 0.95 to 1.1, confirmed the heterozygous nature of the *SEZ6* C>T 27,286,417–27,186,418 substitution.

**Table 2:** Mutant *SEZ6* assay by digital droplet PCR in healthy control subjects, patients with sporadic Alzheimer’s disease, and family members

Healthy population (n = 191)					Sporadic AD cases (n = 9)					Family members (n = 3)					
Sample	Target	Concentration (copies/μl)	Target	Concentration (copies/μl)	Sample	Target	Concentration (copies/μl)	Target	Concentration (copies/μl)	Sample	Target	Concentration (copies/μl)	Target	Concentration (copies/μl)	RATIO (mut/wt)
6	MUT SEZ6	N.D.	WT SEZ6	17	PR3	MUT SEZ6	N.D.	WT SEZ6	239	*PR1*	MUT SEZ6	116	WT SEZ6	103	1.13
7	MUT SEZ6	N.D.	WT SEZ6	14		MUT SEZ6	N.D.	WT SEZ6	226		MUT SEZ6	114	WT SEZ6	120	0.95
8	MUT SEZ6	N.D.	WT SEZ6	11	PR4	MUT SEZ6	N.D.	WT SEZ6	220	PR2	MUT SEZ6	130	WT SEZ6	130	1.00
9	MUT SEZ6	N.D.	WT SEZ6	59		MUT SEZ6	N.D.	WT SEZ6	224		MUT SEZ6	131	WT SEZ6	127	1.03
11	MUT SEZ6	N.D.	WT SEZ6	41	PR6	MUT SEZ6	N.D.	WT SEZ6	267	*PR5*	MUT SEZ6	149	WT SEZ6	153	0.97
12	MUT SEZ6	N.D.	WT SEZ6	49		MUT SEZ6	N.D.	WT SEZ6	261		MUT SEZ6	154	WT SEZ6	152	1.01
13	MUT SEZ6	N.D.	WT SEZ6	36	PR7	MUT SEZ6	N.D.	WT SEZ6	331						
14	MUT SEZ6	N.D.	WT SEZ6	29		MUT SEZ6	N.D.	WT SEZ6	371						
16	MUT SEZ6	N.D.	WT SEZ6	29	PR8	MUT SEZ6	N.D.	WT SEZ6	307						
17	MUT SEZ6	N.D.	WT SEZ6	27		MUT SEZ6	N.D.	WT SEZ6	303						
18	MUT SEZ6	N.D.	WT SEZ6	43	PR9	MUT SEZ6	N.D.	WT SEZ6	254						
19	MUT SEZ6	N.D.	WT SEZ6	30		MUT SEZ6	N.D.	WT SEZ6	266						
21	MUT SEZ6	N.D.	WT SEZ6	35	PR10	MUT SEZ6	N.D.	WT SEZ6	239						
22	MUT SEZ6	N.D.	WT SEZ6	53		MUT SEZ6	N.D.	WT SEZ6	273						
23	MUT SEZ6	N.D.	WT SEZ6	37	PR11	MUT SEZ6	N.D.	WT SEZ6	233						
24	MUT SEZ6	N.D.	WT SEZ6	45		MUT SEZ6	N.D.	WT SEZ6	228						
25	MUT SEZ6	N.D.	WT SEZ6	32	PR12	MUT SEZ6	N.D.	WT SEZ6	212						
27	MUT SEZ6	N.D.	WT SEZ6	26		MUT SEZ6	N.D.	WT SEZ6	190						
28	MUT SEZ6	N.D.	WT SEZ6	47											
29	MUT SEZ6	N.D.	WT SEZ6	48											
30	MUT SEZ6	N.D.	WT SEZ6	30											
34	MUT SEZ6	N.D.	WT SEZ6	30											
36	MUT SEZ6	N.D.	WT SEZ6	49											
38	MUT SEZ6	N.D.	WT SEZ6	32											
39	MUT SEZ6	N.D.	WT SEZ6	34											
41	MUT SEZ6	N.D.	WT SEZ6	74											
42	MUT SEZ6	N.D.	WT SEZ6	43											
44	MUT SEZ6	N.D.	WT SEZ6	53											
46	MUT SEZ6	N.D.	WT SEZ6	64											
51	MUT SEZ6	N.D.	WT SEZ6	55											
52	MUT SEZ6	N.D.	WT SEZ6	19											
53	MUT SEZ6	N.D.	WT SEZ6	32											
60	MUT SEZ6	N.D.	WT SEZ6	46											
61	MUT SEZ6	N.D.	WT SEZ6	44											
62	MUT SEZ6	N.D.	WT SEZ6	64											
64	MUT SEZ6	N.D.	WT SEZ6	55											
66	MUT SEZ6	N.D.	WT SEZ6	45											
67	MUT SEZ6	N.D.	WT SEZ6	46											
69	MUT SEZ6	N.D.	WT SEZ6	48											
70	MUT SEZ6	N.D.	WT SEZ6	45											
71	MUT SEZ6	N.D.	WT SEZ6	67											
72	MUT SEZ6	N.D.	WT SEZ6	57											
74	MUT SEZ6	N.D.	WT SEZ6	54											
89	MUT SEZ6	N.D.	WT SEZ6	47											
90	MUT SEZ6	N.D.	WT SEZ6	78											
91	MUT SEZ6	N.D.	WT SEZ6	64.3											
101	MUT SEZ6	N.D.	WT SEZ6	283											
112	MUT SEZ6	N.D.	WT SEZ6	524											
113	MUT SEZ6	N.D.	WT SEZ6	1451											
114	MUT SEZ6	N.D.	WT SEZ6	962											
115	MUT SEZ6	N.D.	WT SEZ6	534											
118	MUT SEZ6	N.D.	WT SEZ6	527											
119	MUT SEZ6	N.D.	WT SEZ6	1691											
120	MUT SEZ6	N.D.	WT SEZ6	359											
129	MUT SEZ6	N.D.	WT SEZ6	186											
130	MUT SEZ6	N.D.	WT SEZ6	258											
133	MUT SEZ6	N.D.	WT SEZ6	232											
135	MUT SEZ6	N.D.	WT SEZ6	373											
137	MUT SEZ6	N.D.	WT SEZ6	319											
144	MUT SEZ6	N.D.	WT SEZ6	310											
151	MUT SEZ6	N.D.	WT SEZ6	396											
152	MUT SEZ6	N.D.	WT SEZ6	180											
160	MUT SEZ6	N.D.	WT SEZ6	574											
162	MUT SEZ6	N.D.	WT SEZ6	400											
163	MUT SEZ6	N.D.	WT SEZ6	142											
164	MUT SEZ6	N.D.	WT SEZ6	39											
170	MUT SEZ6	N.D.	WT SEZ6	96											
179	MUT SEZ6	N.D.	WT SEZ6	94											
180	MUT SEZ6	N.D.	WT SEZ6	27											
182	MUT SEZ6	N.D.	WT SEZ6	1406											
184	MUT SEZ6	N.D.	WT SEZ6	1994											
185	MUT SEZ6	N.D.	WT SEZ6	161											
192	MUT SEZ6	N.D.	WT SEZ6	14.5											
193	MUT SEZ6	N.D.	WT SEZ6	1740											
197	MUT SEZ6	N.D.	WT SEZ6	185											
198	MUT SEZ6	N.D.	WT SEZ6	250											
199	MUT SEZ6	N.D.	WT SEZ6	145											
200	MUT SEZ6	N.D.	WT SEZ6	132											
202	MUT SEZ6	N.D.	WT SEZ6	663											
205	MUT SEZ6	N.D.	WT SEZ6	658											
206	MUT SEZ6	N.D.	WT SEZ6	118											
210	MUT SEZ6	N.D.	WT SEZ6	103											
212	MUT SEZ6	N.D.	WT SEZ6	23											
214	MUT SEZ6	N.D.	WT SEZ6	385											
215	MUT SEZ6	N.D.	WT SEZ6	125											
219	MUT SEZ6	N.D.	WT SEZ6	223											
223	MUT SEZ6	N.D.	WT SEZ6	316											
228	MUT SEZ6	N.D.	WT SEZ6	109											
233	MUT SEZ6	N.D.	WT SEZ6	385											
237	MUT SEZ6	N.D.	WT SEZ6	767											
240	MUT SEZ6	N.D.	WT SEZ6	318											
241	MUT SEZ6	N.D.	WT SEZ6	15											
243	MUT SEZ6	N.D.	WT SEZ6	30											
245	MUT SEZ6	N.D.	WT SEZ6	166											
247	MUT SEZ6	N.D.	WT SEZ6	161											
251	MUT SEZ6	N.D.	WT SEZ6	164											
253	MUT SEZ6	N.D.	WT SEZ6	491											
254	MUT SEZ6	N.D.	WT SEZ6	772											
255	MUT SEZ6	N.D.	WT SEZ6	771											
257	MUT SEZ6	N.D.	WT SEZ6	148											
261	MUT SEZ6	N.D.	WT SEZ6	875											
263	MUT SEZ6	N.D.	WT SEZ6	381											
267	MUT SEZ6	N.D.	WT SEZ6	442											
270	MUT SEZ6	N.D.	WT SEZ6	368											
275	MUT SEZ6	N.D.	WT SEZ6	317											
276	MUT SEZ6	N.D.	WT SEZ6	368											
277	MUT SEZ6	N.D.	WT SEZ6	186											
278	MUT SEZ6	N.D.	WT SEZ6	63											
279	MUT SEZ6	N.D.	WT SEZ6	234											
287	MUT SEZ6	N.D.	WT SEZ6	99											
293	MUT SEZ6	N.D.	WT SEZ6	125											
324	MUT SEZ6	N.D.	WT SEZ6	605											
325	MUT SEZ6	N.D.	WT SEZ6	153											
326	MUT SEZ6	N.D.	WT SEZ6	692											
327	MUT SEZ6	N.D.	WT SEZ6	713											
328	MUT SEZ6	N.D.	WT SEZ6	391											
332	MUT SEZ6	N.D.	WT SEZ6	759											
333	MUT SEZ6	N.D.	WT SEZ6	661											
337	MUT SEZ6	N.D.	WT SEZ6	798											
338	MUT SEZ6	N.D.	WT SEZ6	903											
340	MUT SEZ6	N.D.	WT SEZ6	40											
341	MUT SEZ6	N.D.	WT SEZ6	274											
342	MUT SEZ6	N.D.	WT SEZ6	240											
344	MUT SEZ6	N.D.	WT SEZ6	209											
345	MUT SEZ6	N.D.	WT SEZ6	873											
348	MUT SEZ6	N.D.	WT SEZ6	2330											
350	MUT SEZ6	N.D.	WT SEZ6	387											
351	MUT SEZ6	N.D.	WT SEZ6	430											
353	MUT SEZ6	N.D.	WT SEZ6	360											
360	MUT SEZ6	N.D.	WT SEZ6	473											
361	MUT SEZ6	N.D.	WT SEZ6	553											
362	MUT SEZ6	N.D.	WT SEZ6	2470											
363	MUT SEZ6	N.D.	WT SEZ6	889											
366	MUT SEZ6	N.D.	WT SEZ6	1990											
367	MUT SEZ6	N.D.	WT SEZ6	452											
368	MUT SEZ6	N.D.	WT SEZ6	1736											
369	MUT SEZ6	N.D.	WT SEZ6	1436											
375	MUT SEZ6	N.D.	WT SEZ6	588											
376	MUT SEZ6	N.D.	WT SEZ6	544											
377	MUT SEZ6	N.D.	WT SEZ6	623											
401	MUT SEZ6	N.D.	WT SEZ6	803											
404	MUT SEZ6	N.D.	WT SEZ6	494											
406	MUT SEZ6	N.D.	WT SEZ6	200											
407	MUT SEZ6	N.D.	WT SEZ6	482											
408	MUT SEZ6	N.D.	WT SEZ6	105											
409	MUT SEZ6	N.D.	WT SEZ6	3260											
418	MUT SEZ6	N.D.	WT SEZ6	190											
422	MUT SEZ6	N.D.	WT SEZ6	1325											
430	MUT SEZ6	N.D.	WT SEZ6	772											
434	MUT SEZ6	N.D.	WT SEZ6	1233											
435	MUT SEZ6	N.D.	WT SEZ6	1844											
440	MUT SEZ6	N.D.	WT SEZ6	90											
446	MUT SEZ6	N.D.	WT SEZ6	745											
451	MUT SEZ6	N.D.	WT SEZ6	1366											
453	MUT SEZ6	N.D.	WT SEZ6	1185											
454	MUT SEZ6	N.D.	WT SEZ6	2950											
466	MUT SEZ6	N.D.	WT SEZ6	329											
468	MUT SEZ6	N.D.	WT SEZ6	681											
493	MUT SEZ6	N.D.	WT SEZ6	80											
497	MUT SEZ6	N.D.	WT SEZ6	154											
499	MUT SEZ6	N.D.	WT SEZ6	128											
501	MUT SEZ6	N.D.	WT SEZ6	1814											
511	MUT SEZ6	N.D.	WT SEZ6	547											
512	MUT SEZ6	N.D.	WT SEZ6	48.2											
513	MUT SEZ6	N.D.	WT SEZ6	40.8											
514	MUT SEZ6	N.D.	WT SEZ6	1019											
519	MUT SEZ6	N.D.	WT SEZ6	1382											
520	MUT SEZ6	N.D.	WT SEZ6	791											
521	MUT SEZ6	N.D.	WT SEZ6	1858											
522	MUT SEZ6	N.D.	WT SEZ6	2180											
523	MUT SEZ6	N.D.	WT SEZ6	1849											
531	MUT SEZ6	N.D.	WT SEZ6	2110											
532	MUT SEZ6	N.D.	WT SEZ6	3030											
535	MUT SEZ6	N.D.	WT SEZ6	1096											
537	MUT SEZ6	N.D.	WT SEZ6	1941											
538	MUT SEZ6	N.D.	WT SEZ6	78											
539	MUT SEZ6	N.D.	WT SEZ6	917											
542	MUT SEZ6	N.D.	WT SEZ6	1650											
543	MUT SEZ6	N.D.	WT SEZ6	937											
545	MUT SEZ6	N.D.	WT SEZ6	1423											
546	MUT SEZ6	N.D.	WT SEZ6	818											
549	MUT SEZ6	N.D.	WT SEZ6	1196											
550	MUT SEZ6	N.D.	WT SEZ6	716											
558	MUT SEZ6	N.D.	WT SEZ6	845											
567	MUT SEZ6	N.D.	WT SEZ6	724											
570	MUT SEZ6	N.D.	WT SEZ6	765											
571	MUT SEZ6	N.D.	WT SEZ6	2290											
574	MUT SEZ6	N.D.	WT SEZ6	790											
575	MUT SEZ6	N.D.	WT SEZ6	1399											
578	MUT SEZ6	N.D.	WT SEZ6	1293											
580	MUT SEZ6	N.D.	WT SEZ6	947											

*ND* Not detectable

Alzheimer’s disease cases are underlined. For each group, patient code, digital droplet PCR target, and the calculated concentration (copies/μl) are reported. For the last group, the ratio, defined as concentration mutant *SEZ6*/concentration wild-type *SEZ6*, is also reported

### Aβ peptide generation in H4-SW cells

**Fig. 3:**
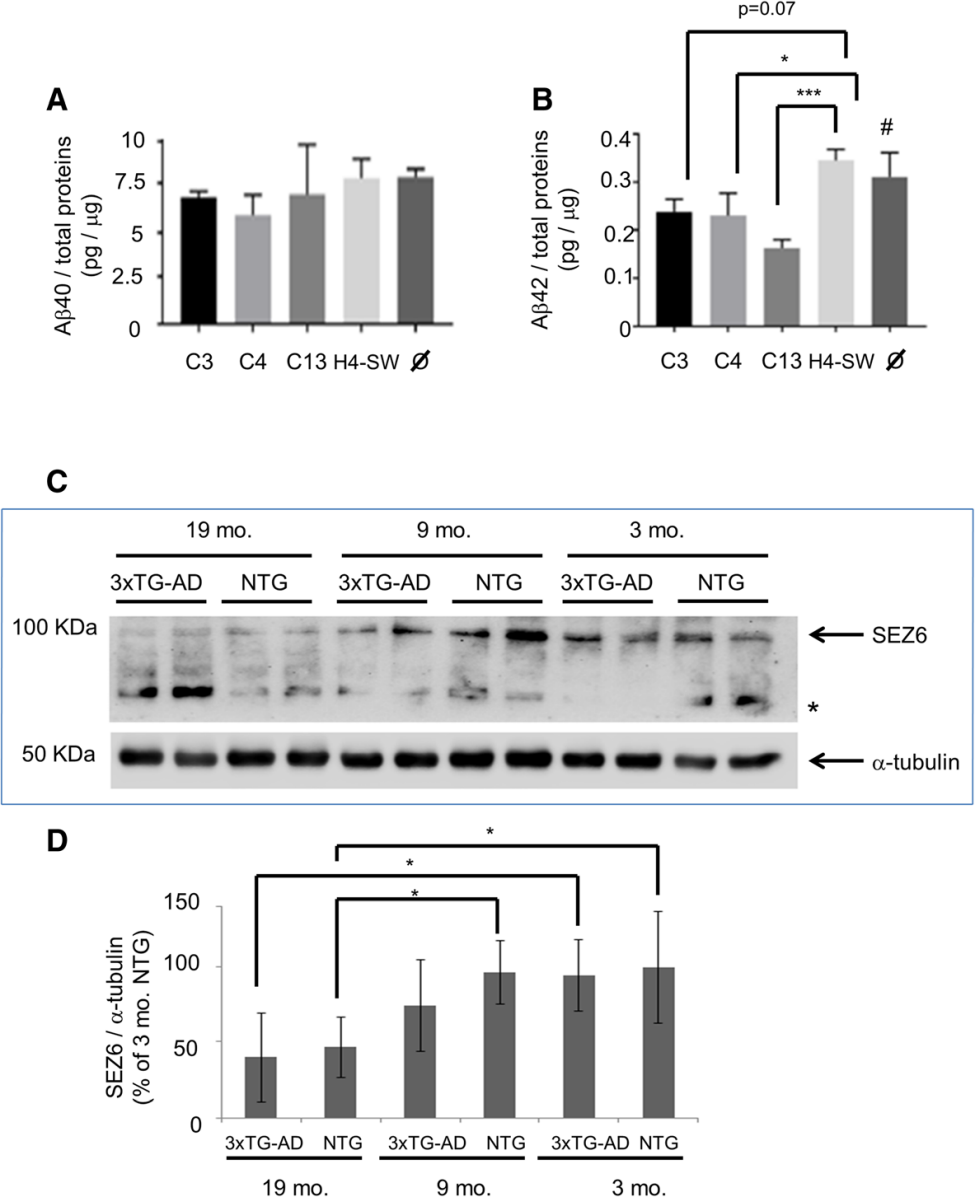
Evaluation of *SEZ6* relevance for Alzheimer’s disease (AD) mechanisms in *in vitro* and *in vivo* models. **a** Quantification by enzyme-linked immunosorbent assay of soluble amyloid-β 1–40 (Aβ_1–40_) in conditioned media from H4-SW clonal lines (C3, C4, and C13) overexpressing *SEZ6*(R615H). The amyloid peptide concentration was normalized to the total protein content of the producing cells of each replicate. Measures are the mean ± SD of three independent wells. *H4-SW* Untransfected control; *Ø* H4-SW control transfected with pCDNA3.1 empty vector. **b** Same as in (**a**) except for the assessment of Aβ_1–42_ soluble form. * *p* < 0.05; *** *p* < 0.001, one-way analysis of variance (ANOVA) and post hoc test; ^#^ *p* < 0.05 vs. C4 and *p* < 0.01 vs. C13, one-way ANOVA and post hoc test. **c** Representative Western blotting for Sez6 protein detection in brain cortical extract from 3xTG-AD mice. Mice were killed at ages 3, 9, or 19 months, and *Sez6* expression was assessed in transgenic and matched nontransgenic (NTG) animals. Each group was composed of three mice, and every animal was loaded in duplicate in the SDS-PAGE experiment. * Unspecific signal. **d** Densitometric quantification of all Western blot analysis data for Sez6 protein cortical expression (*n* = 3 mice/group) using ImageJ software. Each signal was normalized to the corresponding α-tubulin band to control for unequal protein loading. Results are expressed as a percentage of the youngest group (3 months) * *p* < 0.05, one-way ANOVA and post hoc test. *mo.* Months from birth

Three different H4-SW stable clonal lines (C3, C4, and C13) transfected with a pCDNA3.1 plasmid coding for *SEZ6*(R615H) mutant were selected, and the presence of the variant at DNA level was confirmed by allele-specific PCR and sequencing (data not shown). The effect of the R615H substitution on Aβ(1–42) and Aβ(1–40) production by H4-SW cells was assessed in conditioned media from cultured H4-SW(R615H) in comparison to H4-SW cells (untransfected or mock-transfected with an empty pCDNA3.1 vector) (Fig. 3a). The mean concentration of released Aβ(1–42), normalized to cell total protein content, was significantly lower in C4 and C13 than in controls, whereas for the C3 line, there was a trend in the same direction (*p* = 0.07). The Aβ(1–40) assay showed no differences (Fig. 3b).

### *Sez6* brain expression in 3xTG-AD mice

Given that few experimental data linked *SEZ6* to AD, we also examined murine *Sez6* expression in a transgenic line model of AD (3xTG-AD), in comparison with age-matched nontransgenic controls (NTG) (Fig. 3c and d). Mice were killed at ages ranging from 3 to 19 months, and Sez6 protein expression was assessed at brain cortical level. Sez6 protein markedly decreased with age, particularly between 3 and 19 months. However, this reduction was common to both the 3xTG-AD and NTG lines and thus not unique to the AD model.

## Discussion

Pathogenic mutations in *APP*, *PSEN1*, or *PSEN2* genes are linked to FAD [3, 4]. *PSEN1* mutations are responsible for about 60% of the genetic cases of AD, and 286 pathogenic variants have been described in the three above-cited genes [22]. We report an Italian family with AD that we previously screened by denaturing high-performance liquid chromatography (data not shown) for *APP*, *PSEN1,* or *PSEN2* mutations with no results. Considering that rare variants in other genes have been associated with AD [7], we decided to perform targeted exome-sequencing analysis that yielded a large number of variants; in order to identify those closely related to the disease, we employed a recursive filtering strategy. This strategy was based on the removal of high-frequency (> 1%) variants using a public database (1000 Genomes Project) with *in silico* prediction software (SIFT, PolyPhen2, CADD) to exclude potentially harmless mutations and focus on variants present in FAD but not sporadic AD samples. We gave priority to the *SEZ6*(R615H) variant among those reported in Table 1, considering that *SEZ6* has already been reported as relevant for molecular mechanisms involved in AD pathogenesis, because it is a substrate of the BACE-1 enzyme (β-secretase), affects synapse formation, and is reduced in the cerebrospinal fluid of patients with AD, as revealed by a proteomic study [23–25]. *SEZ6* gene mutations have been also reported in association with febrile seizures, and *SEZ6* was proposed as a candidate gene for epilepsy [26, 27]. Moreover, *SEZ6* mutations were found in cases of childhood-onset schizophrenia [28]. The rare variant R615H (rs371753097, C/T) was reported in the 1000 Genomes Project as absent in Toscani in Italy (TSI) population and had a frequency in the whole project of 0.0002 [29]. Another interesting genetic variant we found by exome sequencing that is deserving of attention is A1131P in the *KANK1* gene [30], which was present in the two AD cases (PR1 and PR5) and in PR3, sibling of PR1. However, PR3 did not have dementia at sampling (age 67 years), and her clinical state is currently unchanged, even though we are not able to exclude a possible later onset. The human *KANK1* gene (alias ANKRD15) was originally described to be a tumor suppressor in renal cell carcinoma, and it encodes an ankyrin repeat domain-containing protein (Kank). It belongs to a family of four homologous members that have a role in actin stress fiber formation and renal pathophysiology [31, 32]. There is no reported interaction of *KANK1* with *SEZ6* or AD-related genes. However, a role of *KANK1* mutation or deletion was reported in cerebral palsy spastic quadriplegic type 2, a central nervous system developmental disorder [33]. Moreover, to the best of our knowledge, no data associate *KANK1* with AD.

In our study’s family, we were able to correlate the AD pathology to R615H presence, which was found in the two available AD-affected members and one first-degree relative of an AD case, whose age at sampling in 2003 (PR2, 37 years) was far below the family age of onset (range, 60–70 years) to expect clinical signs. The current clinical diagnosis of PR2 (51 years) is unchanged. We also confirmed that R615H frequency is very low (< 1%) in the Italian population, because we were unable to detect the variant in 200 family-unrelated subjects.

Because it is a common finding that AD pathogenic mutations increase Aβ(1–42) peptide generation [34], we examined the effect of the R615H variant in a cell model in this respect. In the H4-SW line, we noticed a decrease in Aβ(1–42), whereas Aβ(1–40) was unchanged. However, the increase of Aβ(1–42) in association with FAD-linked mutations is not always replicated. In fact, some presenilin mutants with proved pathologic action did not increase Aβ(1–42) but acted on other Aβ peptide generation or even had no impact on this proteolytic cleavage. In the latter case, the hypothesis is that the mutation affects important functions of presenilin other than the γ-secretase activity [35, 36]. It is worth underlining that we found a peculiar biochemical effect of the *PSEN1* mutation E318G that increased Aβ(1–40) only in cultured skin primary fibroblasts [17]. Our failure to detect an increase of Aβ(1–42) might depend on the reported role of SEZ6 protein as substrate for BACE-1 [23], so its overexpression may be competitive for *APP* in the cell model tested. We need further experiments to clarify the role of the R615H variant in this context.

Finally, we followed *SEZ6* cortical expression in a mouse model of AD (3xTG-AD). Considering that it changed similarly in 3xTG-AD and control mice, we were unable to link this result to AD-specific patterns, but we did notice a decrease of SEZ6 protein with age, in agreement with this gene’s reported role in brain development [37, 38]. A damaging mutation (as R615H is predicted to be) may have an impact on the protein activity from birth, with possible neuropathologic outcomes, likely in combination with other triggering factors, also considering the reported role of *SEZ6* in dendritic spine dynamics and cognition [39].

This study has limitations, mainly linked to the unavailability of genomic DNA from all the family’s AD-affected members alive at sampling. Moreover, we decided to use a targeted exome-sequencing strategy that, on one hand, gave us clinical data supporting a rational choice of candidate variants to be prioritized, but on the other hand, prevented us from ruling out that additional coding mutations in genes not included in our panel may be linked to AD phenotype, thus acting in synergy with *SEZ6 (R615H)*.

## Conclusions

In summary, by using a targeted exome-sequencing approach, we discovered a rare *SEZ6* variant exclusive to AD members of a large Italian family carrying no typical FAD-linked mutations that might have a role in disease onset, in particular taking into account the already described involvement of *SEZ6* in AD pathogenic mechanisms linked to amyloid-β (A4) precursor protein (*APP*) and brain physiology, even though the exact molecular pathway linking *SEZ6* to AD is still unclear.

## Additional file

Additional file 1: Table reporting the sequencing results of DNA from PR family members and unrelated sporadic AD cases, including only rare variants in the European population (frequency less than 1%) [low frequency page]. The same reults were further filtered to show variants with predicted damaging action [predicted damage page]. (XLSX 85 kb)
